# An In Vitro Study Demonstrating the Significance of Acromioclavicular Ligament Repair in Restoring Horizontal and Rotational Acromioclavicular Joint Stability

**DOI:** 10.7759/cureus.57193

**Published:** 2024-03-29

**Authors:** Jonathan France, Shantanu Shahane, Apurv Sinha, Ganesh Prasad

**Affiliations:** 1 Orthopaedics, Chesterfield Royal Hospital, Chesterfield, GBR; 2 Trauma and Orthopaedics, Chesterfield Royal Hospital, Chesterfield, GBR

**Keywords:** vertical stability, coracoclavicular reconstruction, rotational stability, horizontal pivot angle, horizontal stability, acromioclavicular repair, acromioclavicular joint reconstruction

## Abstract

Background: The principle of joint reconstruction surgery is to try to recreate the native joint biomechanics and stability. With respect to acromioclavicular joint (ACJ) surgery, much focus to date has been on restoring the superoinferior stability. There is concern that persistent horizontal instability following ACJ reconstruction could lead to poorer patient outcomes; therefore, we evaluated whether acromioclavicular (AC) ligament repair offers improved horizontal stability in conjunction with ACJ reconstruction.

Methods: A whole-body human cadaver was used. The ACJ was exposed and subjected to a constant 70N load on the lateral end of the clavicle to test the anteroposterior (AP), superoinferior (SI), and horizontal pivot angle (HPA) around the ACJ. The AC and coracoclavicular (CC) ligaments were subsequently divided, and the above three parameters were re-tested. Ligament augmentation and reconstruction system (Corin Group, Cirencester, UK), LockDown (LockDown Medical Limited, Redditch, UK), Endobutton (Smith and Nephew Inc., London, UK), and Neoligament implant (Xiros Ltd., Leeds, UK) were used to reconstruct the CC ligaments and tested with and without AC repair.

Results: The native ACJ allowed an average 2.48 mm AP and 3.88 mm SI translation with a 27° HPA. All synthetic implants significantly improved the vertical stability of the ACJ but allowed up to a four-fold increase in AP translation. Coupled with ACJ repair, all the reconstructions were far superior, especially in restoring horizontal stability.

Conclusion: The implants varied in their approach to fixation and concentrated primarily on the reconstruction of CC ligaments. Our study was able to demonstrate that AC repair significantly improves the stability of the construct and significantly reduces vertical and horizontal instability.

## Introduction

Acromioclavicular joint (ACJ) injuries constitute approximately 9% of shoulder girdle injuries [1]. The most widely used classification is that of Rockwood et al., published in 1984, a purely radiological classification. High-grade separations (Rockwood IV-VI) are likely to require surgical stabilisation, but the management of type III injuries remains controversial [2].

There are a number of described techniques for ACJ reconstruction, but there is insufficient evidence as to which technique provides the greatest stability and outcome. Traditionally, the focus has been on restoring the vertical stability of the ACJ, but there is rising clinical evidence that persistent post-operative horizontal instability is associated with inferior functional outcome scores [3].

Horizontal instability is also shown to have a closer association with pain and functional impairment than vertical instability [4-6]. Therefore, a greater emphasis is now placed on ensuring both horizontal and vertical stability when opting for surgical treatment for ACJ injuries [7-9]. There are a number of described surgical techniques that try to recreate the normal anatomy and biomechanics of the ACJ, and so the purpose of this study was to analyse the horizontal and vertical stability following in vitro cadaveric ACJ reconstruction using four commonly used reconstruction techniques.

## Materials and methods

**Figure 1:**
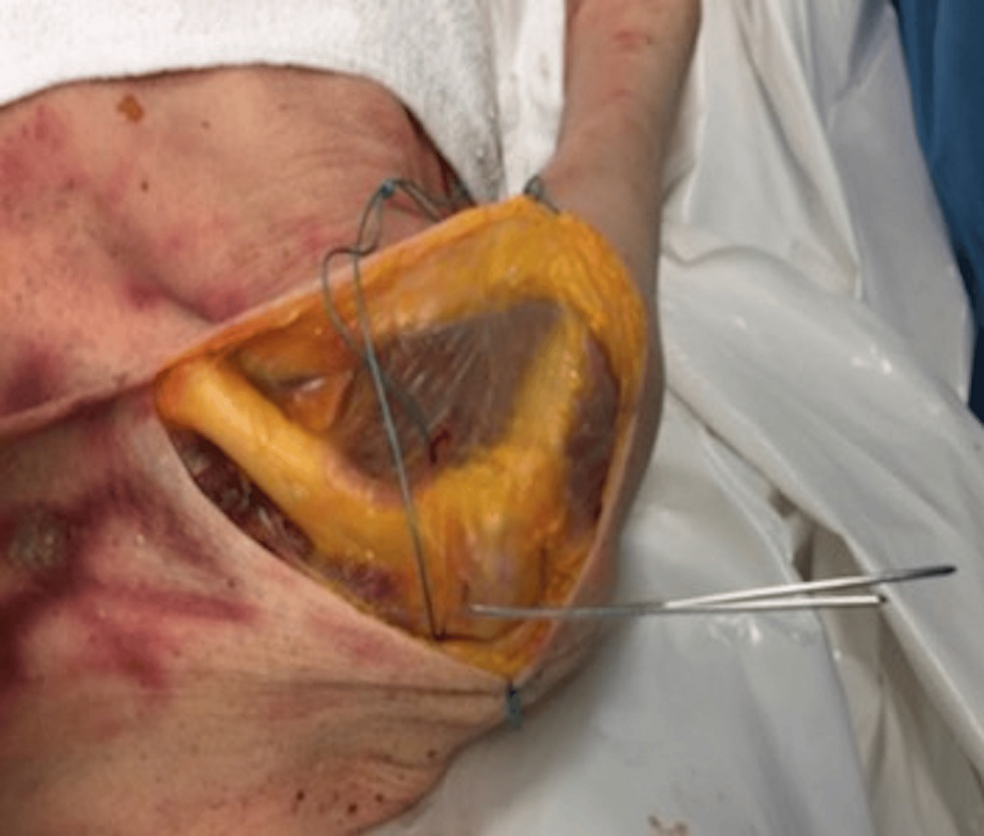
Cadaveric dissection of ACJ with K-wires in the lateral clavicle and acromion for measuring instability in situ ACJ: acromioclavicular joint

This study was performed on one shoulder of a full human cadaver in a controlled environment. The ACJ was exposed, leaving the native acromioclavicular (AC) and coracoclavicular (CC) ligaments intact. Two 1.6 mm K-wires were inserted, one in the acromion and one in the lateral end of the clavicle, to work as fixed bony landmarks for accurate measurements. The native joint was then subjected to a constant maximum load of 70 N (based on previous cadaveric studies using a 50-70 N load) [10,11] on the lateral end of the clavicle using a graduated spring weight. The wire configuration can be seen in Figure 1.

Measurements were taken with an electronic vernier calliper to quantify the anteroposterior (AP) and superoinferior (SI) translations. To measure rotation (horizontal pivot angle (HPA)), the shoulder was moved from a 90-degree abducted position to a flexed, adducted, and internally rotated position (cross-arm adduction). The AC and CC ligaments were sequentially divided, and all the above three parameters were tested again.

In this study, four commonly used synthetic implants: ligament augmentation and reconstruction system (LARS) (Corin Group, Cirencester, UK), LockDown (LockDown Medical Limited, Redditch, UK), Endobutton (Smith and Nephew Inc., London, UK), and Neoligament implant (Xiros Ltd., Leeds, UK) were used to reconstruct the CC ligaments in accordance with the technique described by the manufacturers and tested with and without AC ligament repair (interosseous horizontal mattress No. 2 Ethibond Excel Polyester Suturesuture (Ethicon, Johnson and Johnson Medical Devices, Belgium). These were chosen as the authors felt that they represented and covered the wide variety of synthetic implants and configurations of reconstructions available at the time of the study, with Table 1 highlighting the main differences between them.

**Table 1:** Comparison of the four ACJ reconstruction techniques used in this study LARS: ligament augmentation and reconstruction system; ACJ: acromioclavicular joint

Fixation device	Coracoid attachment	Clavicular attachment	Number of limbs	Direction of limb/plane
LARS	Passed around the neck	Two inter-osseous holes with interference screw	2 to 4 (figure of 8)	Oblique V configuration covering sagittal and coronal plane
LockDown	Passed around the neck	Passed around the clavicle with screw fixation of the end	1	Oblique in sagittal plane
Endobutton	Interosseous drill hole through the coracoid	Interosseous drill hole through clavicle	1	Straight
Neoligament	Passed around the neck	Interosseous drill hole through clavicle	1 or multiple if required	Straight or multiple

The ligaments available varied in their approach to CC ligament repair. At the coracoid, fixation options are to go around/loop the neck/base of the coracoid or to go through the coracoid with a drill hole. On the clavicle side, the options are to loop around or go through single or multiple drill holes in different planes. The LARS ligament is looped around the coracoid base, then passed through two holes in the clavicle in different planes to match the anatomy of the conoid and trapezoid ligaments, and secured with two interference screws for each limb. Residual length can be looped around the coracoid or acromion for added stability if required.

LockDown ligament is looped around the coracoid and then passed over the clavicle from posterior to anterior and secured in position with a single screw through the loop-tip of the ligament. It offers a single-limb construct. The Endobutton has a similar fixation technique on both sides, where a drill hole is used through the coracoid base and clavicle with metallic buttons on either side.

The Neoligament is looped around the coracoid like LockDown, but then its limbs are passed through a single drill hole in the clavicle and secured with a knot over a metallic button. Residual length can be looped around the coracoid or acromion for added stability if required.

## Results

The native joint with an intact ACJ allowed an average of 2.48 mm of AP and 3.88 mm of SI translation with 27° rotation (HPA) around the ACJ in cross-arm adduction. After the AC ligaments were divided, there was an average of 4.07 mm of AP and 9.78 mm of SI translation. With the division of CC ligaments, gross instability was noted with an average AP translation of 20.96 mm and SI translation of 23.69 mm. Table 2 below shows the outcomes of the different reconstruction techniques with and without ACJ repair compared to the native joint.

**Table 2:** Outcomes of the different reconstruction techniques with and without ACJ repair ACJ: acromioclavicular joint; CC: coracoclavicular; LARS: ligament augmentation and reconstruction system

	Anteroposterior (mm)	Superoinferior (mm)	Horizontal pivot (degrees)
Native	2.48	3.88	27
ACJ dissected	4.07	9.78	23
CC dissected	20.96	23.69	29
LARS 8	5.3	5.39	21
LARS 8 + ACJ	3.24	4.7	20
Lockdown	5.7	5.45	27
Lockdown + ACJ	3.35	2.01	19
Endobutton	10.54	5.52	24
Endobutton + ACJ	3.04	0.87	19
Neoligament	11.91	2.36	23
Neoligament + ACJ	1.65	0.21	16

All synthetic ligament implants improved the vertical stability of the ACJ, allowing approximately 5 mm of movement in this plane, close to that of the native joint. However, when it came to horizontal stability, ACJ reconstruction in isolation permitted residual movement between two and four times what the native joint allows. 

When ligaments were tested alone without an ACJ capsuloligamentous repair, single-limb ligaments (Neoligament and Endobutton) traveling straight from clavicle to coracoid performed very well in the vertical (SI) plane but allowed greater translation in the horizontal (AP) plane. Multiple-limb ligaments (LARS) and obliquely running LockDown both have multiple plane configurations and were found to have improved stability in both the SI and AP planes.

When coupled with ACJ capsuloligamentous repair, all the reconstructions were superior in all the parameters of measurement, especially horizontal (AP) instability, including single-limb repairs (Neoligament and Endobutton). The combination of AC and CC ligament reconstruction was superior to any reconstruction in isolation and did not depend on the type of ligament used to reconstruct the CC ligaments. None of the reconstructions failed under the load (70 N) applied.

## Discussion

Various biomechanical studies have been reported in the medical literature, emphasising the significance of AC ligament repair to restrain posterior axial rotation and therefore restore horizontal and rotational ACJ stability. The majority of these studies refer to the pioneering work on describing ACJ anatomy by Urist [12] and Fukuda et al. [7]. An in vitro study on synthetic bone models by Holzer et al. [13] concluded that a figure of eight was the most stable construct to restore horizontal and rotational ACJ stability. Luis et al. [14], in their study of 54 cadavers, laid emphasis on the significance of augmenting the modified Weaver-Dunn procedure with AC capsule-ligamentous repair to restore horizontal stability. Saier et al. cyclically loaded 12 cadaveric shoulders and demonstrated the value of additional AC cerclage for horizontal stability in ACJ separation [15]. Mazzocca et al. [4] evaluated the three-dimensional movement of 24 cadaveric shoulders with digitised cameras to conclude that reconstruction of the AC ligaments was the most stable method of restoring rotation comparable to the native joint. In 2017, following a biomechanical study on 24 cadaveric shoulders, Beitzel et al. [3] concluded that an anatomical repair should address both CC and AC ligaments to restore physiologic function (rotation and translation).

More recently, Lee et al. [10] conducted a cadaveric study to better understand the ACJ’s complex biomechanical characteristics and aid in the development and evaluation of reconstruction techniques. Their most important finding was that the ACJ capsule was the principal restraint to anterior and posterior displacement of the distal clavicle, clearly dominating instability in the inferior direction but not providing significant resistance to superior displacement. The conoid ligament was dominant in preventing superior displacement and shared load with the ACJ capsule in anterior and posterior directions.

In managing 46 acute ACJ dislocations, Lizaur et al. [16] demonstrated that repair of the CC ligaments had no bearing on the final stability of the clavicle and that supraclavicular enforcement by suture and overlap of the deltoid and trapezius were sufficient. The ACJ was protected with K-wires for four to five weeks. Seventy-four percent had no loss of reduction, with 91% being very satisfied with their function. Clinically superior results were demonstrated with the arthroscopic stabilisation of chronic ACJ instabilities with the GraftRope technique with additional horizontal tendon augmentation by Saier et al. [15]. Further, Scheibel et al. [9], in their study of 28 arthroscopically treated patients, reported that evidence of posterior instability had significantly inferior results. Recently, Liu et al. [17] described ACJ reconstruction with two horizontal No. 2 Ethibond Excel Polyester Suture across the ACJ for horizontal stability and two suture anchors to repair the conoid and trapezoid ligaments for vertical stability. They published a retrospective review of 29 patients with a mean follow-up of 28 months and reported statistically significant clinical results with no horizontal instability in any of their patients.

In our study, we observed that CC ligament reconstruction using LARS 8 configuration, LockDown, Endobutton, and Neoligament improved vertical ACJ instability but without ACJ ligament repair, allowing two to four times more translation than the native joint in the horizontal plane. The type of ligament used and the configuration used did not matter, and all implants had improved horizontal stability once the intra-osseous AC ligament repair was done. This combined augmented repair closely resembled the native joint in all parameters of measurements.

The authors understand there were limitations to this study. This was an in vitro cadaveric study without any active dynamic musculature to account for the forces acting on a shoulder. Additionally, we cannot ascertain the degree of residual movement at the ACJ that would be clinically significant due to the paucity of prior studies. The authors have taken the initial measurements from the native joint as a standard. Only the four most commonly available and widely used methods of fixation have been tested in this study. Non-anatomic and biological repairs like the Weaver-Dunn procedure and hamstring repairs have not been tested.

## Conclusions

The synthetic ligament implants varied in their approach to fixation and concentrated primarily on the reconstruction of CC ligaments. Our study was able to demonstrate that AC ligament repair significantly improves the stability of the construct and significantly reduces vertical (SI) and horizontal (AP) instability. This surgical technique closely replicates the native joint in vitro, which none of the available synthetic implants were able to achieve on their own. Based on the strong observations in this in vitro pilot study, further exploration into the clinical significance of these findings is warranted.

## References

[1] Mazzocca AD, Arciero RA, Bicos J (2007). Evaluation and treatment of acromioclavicular joint injuries. Am J Sports Med.

[2] Rockwood CA Jr, Williams GR Jr, Young DC (2004). Disorders of the acromioclavicular joint. The shoulder 3rd Ed.

[3] Cisneros LN, Reiriz JS (2017). Prevalence of remaining horizontal instability in high-grade acromioclavicular joint injuries surgically managed. Eur J Orthop Surg Traumatol.

[4] Fukuda K, Craig EV, An KN, Cofield RH, Chao EY (1986). Biomechanical study of the ligamentous system of the acromioclavicular joint. J Bone Joint Surg Am.

[5] Jensen G, Katthagen JC, Alvarado L, Lill H, Voigt C (2013). Arthroscopically assisted stabilization of chronic AC-joint instabilities in GraftRope™ technique with an additive horizontal tendon augmentation. Arch Orthop Trauma Surg.

[6] Scheibel M, Dröschel S, Gerhardt C, Kraus N (2011). Arthroscopically assisted stabilization of acute high-grade acromioclavicular joint separations. Am J Sports Med.

[7] Beitzel K, Obopilwe E, Apostolakos J (2014). Rotational and translational stability of different methods for direct acromioclavicular ligament repair in anatomic acromioclavicular joint reconstruction. Am J Sports Med.

[8] Mazzocca AD, Conway JE, Johnson S, Rios CG, Dumonski ML, Santangelo SA, Arciero RA (2004). The anatomic coracoclavicular ligament reconstruction. Op Tech in Sports Med.

[9] Tauber M (2013). Management of acute acromioclavicular joint dislocations: current concepts. Arch Orthop Trauma Surg.

[10] Lee J, El-Daou H, Alkoheji M, Carlos A, Di Mascio L, Amis A (2021). Ligamentous and capsular restraints to anterior-posterior and superior-inferior laxity of the acromioclavicular joint: a biomechanical study. J Shoulder Elbow Surg.

[11] Banffy MB, Uquillas C, Neumann JA, ElAttrache NS (2018). Biomechanical evaluation of a single- versus double-tunnel coracoclavicular ligament reconstruction with acromioclavicular stabilization for acromioclavicular joint injuries. Am J Sports Med.

[12] Urist MR (1946). Complete dislocations of the acromiclavicular joint; the nature of the traumatic lesion and effective methods of treatment with an analysis of forty-one cases. J Bone Joint Surg Am.

[13] Holzer N, Cunningham G (2017). Technique for acromioclavicular stabilisation effects rotational stability of the acromioclavicular joint. 18th EFORT Congress.

[14] Luis GE, Yong CK, Singh DA, Sengupta S, Choon DS (2007). Acromioclavicular joint dislocation: a comparative biomechanical study of the palmaris-longus tendon graft reconstruction with other augmentative methods in cadaveric models. J Orthop Surg Res.

[15] Saier T, Venjakob AJ, Minzlaff P (2015). Value of additional acromioclavicular cerclage for horizontal stability in complete acromioclavicular separation: a biomechanical study. Knee Surg Sports Traumatol Arthrosc.

[16] Lizaur A, Marco L, Cebrian R (1994). Acute dislocation of the acromioclavicular joint. Traumatic anatomy and the importance of deltoid and trapezius. J Bone Joint Surg Br.

[17] Liu T, Bao FL, Jiang T, Ji GW, Li JM, Jerosch J (2020). Acromioclavicular joint separation: repair through suture anchors for coracoclavicular ligament and nonabsorbable suture fixation for acromioclavicular joint. Orthop Surg.

